# Use of a Percutaneous Right Ventricular Assist Device to Manage Right Ventricular Failure in Inferior STEMIs

**DOI:** 10.7759/cureus.52735

**Published:** 2024-01-22

**Authors:** Sushant Koirala, Michael Sunnaa, Mina M Kerolos, Hussam Suradi

**Affiliations:** 1 Internal Medicine, RUSH University Medical Center, Chicago, USA; 2 Cardiology, University of Chicago Medical Center, Chicago, USA; 3 Cardiology/Interventional Cardiology, RUSH University Medical Center, Chicago, USA

**Keywords:** impella rp, intra-aortic balloon pump, acute st elevation myocardial infarction, acute right ventricular failure, rvad

## Abstract

Acute right heart failure is a complication of inferior ST-elevation myocardial infarctions. Given the further hemodynamic instability that results from right-sided failure, a treatment option is needed to help bridge toward cardiac recovery. We present a case of using a right ventricular assist device in a patient who had marked improvement in cardiac function after an instance of acute right heart failure.

## Introduction

Acute right heart failure (RHF) is a rare but life-threatening complication that can occur following an inferior ST-elevation myocardial infarction (STEMI). While the management of left ventricular dysfunction in STEMI has been extensively studied, acute RHF poses unique challenges with limited standardized therapeutic options. The use of mechanical circulatory support devices has emerged as a promising strategy to address this critical clinical scenario. In this case report, we present a compelling instance of the successful deployment of the Impella® RP (Abiomed, MA) device in a patient experiencing acute RHF after an inferior STEMI. This case was previously presented as a poster at the 2023 ACC Midwest Cardiovascular Forum on November 4, 2023.

## Case presentation

A 62-year-old male with a past medical history of diabetes, hypertension, and dyslipidemia initially presented to the emergency department via emergency medical service (EMS) for an inferior wall ST-elevation myocardial infarction (STEMI) with a complete heart block (CHB). A CODE STEMI was activated for planned percutaneous coronary intervention (PCI). Upon arrival at the emergency department, the patient was found to be hypotensive and bradycardic. He was started on intravenous (IV) fluids, atropine pushes, and a dopamine infusion to maintain heart rate and blood pressure.

The patient briefly coded after a likely vagal episode triggered by nausea/vomiting but achieved a return of spontaneous circulation (ROSC) with less than 30 seconds of cardiopulmonary resuscitation (CPR). During the transfer to the cath lab, the patient once again became substantially bradycardic, necessitating another code blue. The patient underwent rapid sequence intubation and arrived at the cath lab, where a right femoral vein access was established for a temporary transvenous pacemaker because of his high-grade atrioventricular (AV) block. The right femoral artery was accessed for the PCI.

The patient was found to have severe single-vessel coronary artery disease, with 100% stenosis at the right coronary ostium, along with flush occlusion of the right coronary artery (RCA). The thrombolysis in myocardial infarction (TIMI) grade was 0, with no flow across the lesion. Given the size and degree of the disease in the RCA, the patient underwent three lesion interventions with balloon angioplasty followed by drug-eluting stent placement. Post-intervention, the lesion exhibited 0% residual stenosis with TIMI grade III flow. An intra-aortic balloon pump was also placed, and the patient was started on a heparin drip before being transferred to the cardiac ICU for further management.

Upon arrival at the cardiac intensive care unit, the patient remained intubated, with the balloon pump operating at a 1:1 ratio and a transvenous pacer in place. All extremities were cool to the touch, and PT pulses were nonpalpable but detectable with Doppler. The patient remained hypotensive and received a 1 L bolus with a good response, followed by initiation of a dopamine drip. A bedside echo revealed severe right ventricular hypokinesis and dilation, with a hyperdynamic left ventricle. Central venous pressure was significantly elevated at 24, with pulmonary artery (PA) pressures of 33/26/29 and pulmonary capillary wedge pressure (PCWP) of 25.

Because of persistent hypotension and Swan-Ganz measurements suggesting right ventricular (RV) failure secondary to the inferior STEMI, the decision was made to perform an emergent Impella RP® placement in the cath lab. A repeat echocardiogram post-device placement still showed right ventricular dilation and diminished systolic function; however, there was an improvement compared to the bedside echocardiogram (Video 1). There was also an improvement in the left ventricular ejection fraction. Central venous pressure significantly improved to nine after device placement.

**Video 1:** Complete transthoracic echocardiogram done immediately post-Impella RP® placement

The patient required continuous inotropic support with dobutamine, as well as vasopressor support with norepinephrine and vasopressin for a period of time. Dobutamine was eventually weaned, and the balloon pump was transitioned to a 1:3 ratio without changes in mixed venous oxygenation saturation. Three days after Impella® placement, the intra-aortic balloon pump was removed without issues. The following day, the Impella® device was also removed, and a repeat EKG showed a resolution of complete heart block, prompting the removal of the transvenous pacer.

The patient underwent aggressive diuresis based on Swan-Ganz readings. Because of clinical improvement, the Swan-Ganz catheter was removed seven days after transfer to the cardiac intensive care unit. The final echocardiogram before discharge showed a recovered left ventricular ejection fraction of 60 to 65%, with mild hypokinesis of the basal/mid myocardium and hyperdynamic left ventricular systolic function.

## Discussion

While cardiogenic shock secondary to acute RV failure is frequently associated with unfavorable outcomes and elevated mortality rates, small-scale retrospective studies and case series have indicated that the RV possesses the potential for rapid recovery and demonstrates greater resistance to ischemic injury compared to the left ventricle [1]. Right ventricular assist devices (RVADs) such as Impella RP® have demonstrated the ability to rapidly stabilize hemodynamics, improve RV function, and increase cardiac output, leading to a swift reversal of shock and improved survival [2]. Furthermore, there have been case series and small-scale studies that have shown that the use of the Impella RP® device is associated with reduced mortality rates and improved end-organ perfusion [3]. Although the use of RVAD devices such as the Impella RP® appears promising, they do come with risks, including bleeding, hemolysis, and vascular complications. Furthermore, there has been no large-scale randomized trial to study the use of percutaneous RVAD devices because of their infrequent utilization. However, in the limited single-center studies that have been conducted, the use of these devices has shown that despite the expected device-related complications, a higher survival rate than the nationally reported rate in patients with acute RV dysfunction was found [4,5].

## Conclusions

Percutaneous RVAD such as the Impella RP® device is an important therapeutic option for managing acute right ventricular failure in the context of an inferior STEMI. Because of the rate of mortality seen in acute right ventricular failure, prompt response with an RVAD device can significantly decrease this rate of mortality, as well as increase the odds of right ventricular recovery. As promising as the few single-center studies are, however, this topic needs more extensive studies in a randomized setting to fully understand the degree of benefit.
